# Humoral immune response profiling with peptide microarrays

**DOI:** 10.1186/1742-4690-9-S2-P95

**Published:** 2012-09-13

**Authors:** U Reimer, H Wenschuh, LR Baden, M Pau, M Weijtens, DH Barouch

**Affiliations:** 1 JPT Peptide Technologies, Berlin, Germany; 2 Div. of Vaccine Res., Beth Israel Deaconess Medical Center, HMS, Boston, MA, USA; 3 Crucell Holland BV, Leiden, the Netherlands

## Background

In a case-controlled analysis designed to identify immune correlates of infection risk in the RV144 HIV vaccine trial, 2/6 primary variables showed significant correlation. One, the binding of IgG antibodies to the V1/V2 loop of HIV-1 env protein appears to protect against HIV-1 infection. Consequently, data from a detailed mapping of antibody reactivities in response to vaccination on a sub-protein level might be a predictor for vaccine efficacy. In contrast to assays relying on whole antigens, peptide microarrays are efficient tools to deliver such information. Besides, complex peptide libraries can cover the HIV sequence diversity. We present peptide microarray data from a human clinical trial.

## Methods

A library representing Env-gp160 consensus sequences from clades A,B,C,D,M,CRF1, and CRF2 was produced. Serum samples of vaccinees from groups receiving different doses of a prototype Ad26 vector-based vaccine expressing clade A-HIV-1 Env (Ad26.EnvA.01) were evaluated. For the calculation of signals the signal intensity per peptide at baseline was substracted from the signal intensity at week 28 after vaccination.

## Results

All groups of vaccinees show a clear pattern of antibody reactivity after vaccination. This pattern depends on the dose and the number of doses given. From the lowest doses of 1x10^9^ viral particles (vp) a cross-clade reactivity towards the V3 region of gp120 is observed. At doses above 1x10^10^ vp the magnitude of signals is enhanced and new regions of gp120 are targeted by patient antibodies, e.g. towards the V2 loop region. The representation of different clades on the peptide microarray allows for a detailed investigation of the clade specificity of the antibody response after vaccination.

## Conclusion

Costly vaccination studies require consideration of all possible factors for success. The results of peptide microarray experiments may facilitate the design and dosing regimen of vaccines in clinical trials and shed light on the underlying protective mechanisms.

